# Investigation on the Flexural–Tensile Rheological Behavior and Its Influence Factors of Fiber-Reinforced Asphalt Mortar

**DOI:** 10.3390/polym12091970

**Published:** 2020-08-30

**Authors:** Xiaoyuan Zhang, Li Xu, Junxiu Lv

**Affiliations:** 1 School of Civil Engineering and Architecture, Zhejiang Sci-Tech University, Hangzhou 310018, Zhejiang, China; 2 Zhejiang Communications Construction Group Co., Ltd., Hangzhou 310051, Zhejiang, China; 3 College of Traffic and Transportation, Chongqing Jiaotong University, Chongqing 400074, China; 4 School of Transportation, Southeast University, Nanjing 210096, Jiangsu, China; 5 The Architectural Design & Research Institute of Zhejiang University Co., Ltd., Hangzhou 310028, Zhejiang, China; hangzxl@163.com (L.X.); lvjunxiu1112@163.com (J.L.)

**Keywords:** basalt fiber, fiber content, asphalt mortar, flexural–tensile rheological behavior, aspect ratio

## Abstract

On the basis of a modified three-dimensional (3D) random distribution fiber model, this study further investigates the flexural–tensile rheological behavior and its influence factors of fiber-reinforced asphalt mortar. First, the viscoelastic creep at a temperature of 15 °C for pure asphalt mortar as the control sample are obtained by the beam bending creep test to fit the Burgers constitutive parameters. Second, a 3D numerical model consisting of a homogeneous asphalt mortar matrix with viscoelastic parameters and short and straight fibers with elastic characteristics is built in a cuboid space on the basis of a fiber algorithm to simulate the flexural–tensile rheological behavior using ABAQUS software, and the rheological behavior of the 3D model is consistent with those of the test result. Finally, 3D numerical simulations are conducted to further analyze the effect of fiber factors (e.g., contents, aspect ratios, modulus, and fiber types) on the rheological behavior. Results show that the effect of basalt fiber (BF) compared with steel wool fiber are more significant, and increasing fiber contents and aspect ratios have a positive reinforcement effect on the rheological behavior, where BF content for 0.1%, 0.2%, and 0.3% at 3600 s compared with the control reduced by 37.5%, 53%, and 61.7%, and BF aspect ratios for 30, 40, and 50 compared with that for 20 increased by 4.3%, 16.1%, and 32.9%, respectively, but the change in fiber modulus has a minimal impact.

## 1. Introduction

The addition of fiber to asphalt mixtures has been widely studied [1,2,3]. For example, Rust et al. [4] have evaluated the influence of nylon fiber on the anticracking performance of dense asphalt mixtures using laboratory tests; their testing results indicated that fracture energy of dense asphalt mixtures prepared with 1% fiber contents are 85% higher than that of nonreinforced asphalt mixtures. Wu et al. [5] have investigated pavement performances of carbon fiber asphalt mixture through the indirect tensile test; their testing results indicated that the residual stability increases from 91.1% to 92.7% and the Marshall stability from 12.8 to 13.5 kN. Ye et al. [6,7,8] have indicated that adding polyester fiber can effectively enhance the fatigue property of asphalt mixtures under low stress levels. Li et al. [9] have reported an excellent performance regarding the antirutting of diatomite and glass fiber compound modified asphalt mixtures.

Compared with the prevalent fiber additives in asphalt mixtures mentioned above, the use of some high-strength fiber additives, such as adding steel wool fiber (SWF) or basalt fiber (BF), into dense asphalt mixtures have also been examined [10,11,12,13,14,15,16,17]. For instance, García et al. [10] have explored the fiber distribution of SWF and its the electrical conductivity in dense asphalt mixtures; their results showed that thick and short fibers can disperse well in asphalt mixtures to ensure that fibers do not have a relevant influence on the particle loss resistance. Moreover, it was reported that BF had more excellent mechanical performances and durability than plant fiber, and were more environmentally friendly than glass and carbon fibers [18,19,20]. Morova [11] have investigated the influence of BF on asphalt mixtures by the laboratory test; the results showed that the 5% bitumen content under 0.5% fiber contents had better stability than other asphalt contents. Yu et al. [12] have investigated the effect of BF and diatomite compound modified asphalt mixtures using laboratory tests; their results showed that fiber asphalt mixtures could obtain more excellent properties than without fiber asphalt mixtures.

As a polymer composite material, the mechanical behavior of asphalt mixtures largely depends on the asphalt-like components [21]. The influence of stabilizing and reinforcing fibers on the behavior of the asphalt-like components as binding materials in asphalt mixtures have been widely studied [22,23,24]. As a typical binding material, asphalt mortar can be considered as a lower scale of asphalt mixtures, which composed of asphalt mastic (including asphalt binder and filler) and fine aggregate, and exhibits complex thermorheological properties affecting the mechanical behavior of asphalt mixtures. Short fibers added into asphalt mixtures can optimize properties of binding materials, such as stability, reinforcing, anticracking, and toughening, to effectively improve the pavement performances of asphalt mixtures [3,19,25,26,27]. Some investigations also indicated that asphalt mortar containing BFs shows excellent performances such as antirutting and fatigue at different temperatures [28,29,30]. For example, Gu et al. [28] have investigated the rheological behavior and internal mechanism of BF-reinforced asphalt mortar polymer under a high in-service temperature by the numerical simulation analysis and laboratory tests verification, where the distribution and orientation of fibers in the polymer matrix model were set as directional arrangement. Zhang et al. [30] have further researched three-dimensional (3D) mesostructural modeling of BF random orientation in cylindrical or cuboid matrix to get the impact of different fiber distributions (containing random and directional) on the mechanical behavior of asphalt mortar polymers; the simulated results of the 3D fiber random distribution were verified by the relevant experiment. Wang et al. [31] investigated the viscoelastic creep behavior of unidirectional short fiber-reinforced polymer composites by the finite element method, simulated results showed that compared with the creep compliance coefficients along other directions, the coefficient along the direction of fiber arrangement can be more decreased with the increase of fiber aspect ratios.

However, the reinforcement effect of fibers on asphalt mortar polymers was explored by only considering 3D mesostructural distribution at a given fiber content. A limited body of work has further adopted numerical methods to analyze the influence of other fiber factors, including fiber types, contents, aspect ratios, and modulus in composite space, on the flexural–tensile rheological behavior of asphalt mortar polymers at intermediate temperatures. Consequently, scholars must further research the effect of various influence factors on the rheological behavior of fiber-reinforced asphalt mortars (FRAMs) to reveal the internal mechanism and verify the reliability of the 3D random-distribution fiber model.

## 2. Objectives

**Figure 1:**
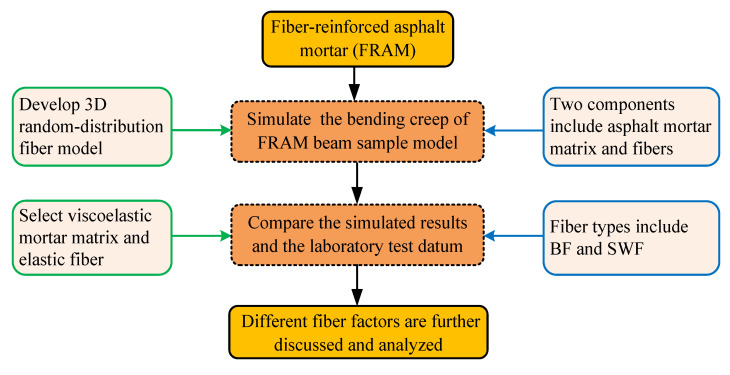
Research plan process.

This study mainly investigated the influence of various fiber factors, such as contents, aspect ratios, modulus, and fiber types on the bending rheological behavior of asphalt mortar polymers on the basis of the 3D random-distribution fiber model. The proposed model is assumed to consist of two parts, e.g., asphalt mortar matrix polymer and short fibers, where the polymer matrix is considered as homogeneous with viscoelastic behavior, and fiber with high strength elastic are randomly dispersed into the matrix. Two kinds of fibers, namely, BF and SWF, were used to analyze the impact of fiber types. The bending creep tests of the FRAM beam sample model under randomly distributed fibers are simulated in the ABAQUS using the developed 3D finite element (FE) model. The numerical results and the test datum are compared, and the other fiber factors in the asphalt mortar material are further analyzed at an intermediate in-service temperature. The research plan is shown in Figure 1.

## 3. Testing and Simulation Method

### 3.1. Materials

The PG76-22 grade SBS-modified bitumen, limestone filler, and limestone fine aggregate were selected as the raw materials of asphalt mortar polymers, and their properties were all satisfied according to the standards JTG E42-2005 and JTG E20-2011 of China [32,33], where the nominal maximum value of fine aggregate particles was less than 2.36 mm. Moreover, asphalt mortar can be considered a binder material in mesoscale levels of the asphalt mixture and was prepared according to the standards JTG F40-2004 of China [30,34]. On the basis of the specific surface area method [30], the bitumen–aggregate ratio of the asphalt mortar for AC13 gradation was obtained at about 13.0%, where the corresponding bitumen–aggregate ratio of the asphalt mixture was 4.9%. Table 1 presented the gradation of the asphalt mixture and the corresponding mortar.

**Figure 2:**
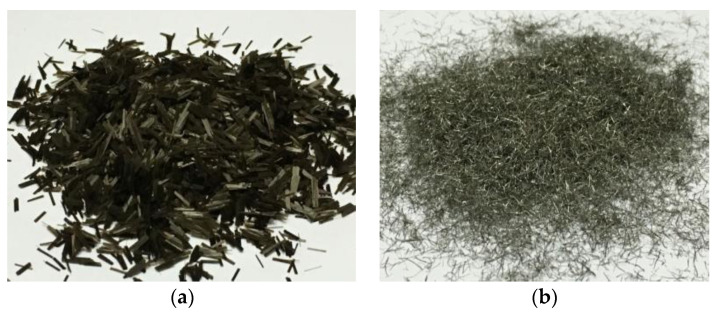
Two kinds of fibers used in asphalt mortar (**a**) basalt fiber (BF) and (**b**) steel wool fiber (SWF).

BF and SWF of 6 mm length were selected [30], where BF and SWF had a diameter of 0.02 and 0.1 mm, as presented in Figure 2a,b. The fiber contents in the asphalt mixture were all selected to 0.3%, corresponding to 0.79% in the asphalt mortar for laboratory tests [30]. The indicators of fibers were listed in Table 2. 

### 3.2. Testing Method

The fiber was positioned into a 105 °C oven for about 4 h to ensure moisture-free surfaces, and then the dry process was adopted to blend the fiber with the fine aggregate before adding the asphalt binder. In order to obtain the influence of fiber on asphalt mortars, two fiber contents (i.e., 0% and 0.79%) in the asphalt mortar were selected, where three of the same samples were prepared under each fiber content. The FRAM beam samples were prepared in molds by a static pressure method, where the size (length × breadth × height) of the beam sample were selected as 160 mm × 40 mm × 40 mm.

**Figure 3:**
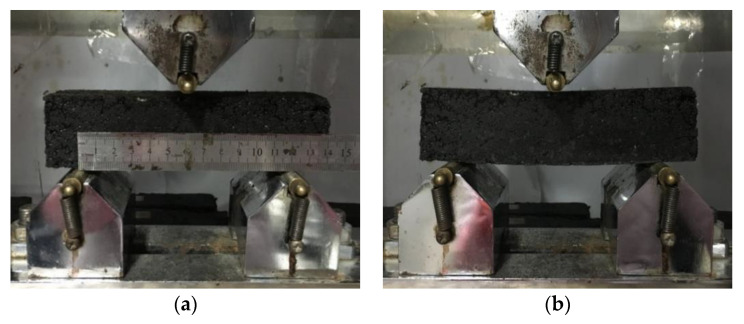
Loading of the fiber-reinforced asphalt mortar (FRAM) beam sample (**a**) before loading and (**b**) loading.

The three-point bending creep test was selected to obtain midspan deflection of FRAM beam samples, where temperature was kept at 15 °C in the UTM-25 (IPC Global, Melbourne, Australia), and the loading time was set to 3600 s under a constant load. Moreover, the center distance of the sample support was 120 mm, and the upper pressure and end support heads were all made of a circular-arc fixed steel bar with 10 mm radius, as shown in Figure 3.

The flexural–tensile stress $\sigma_{0}$ and corresponding strain ε(t) of the beam sample at the midspan bottom are calculated as follows [30]:

(1) $$\sigma_{0}=\frac{3lF_{0}}{2bh^{2}},$$

(2) $$\varepsilon (t)=\frac{6hd(t)}{l^{2}},$$

where *l* indicates the span of the sample, mm; *F*_0_ denotes the constant vertical load at the midspan, N; *b* is the cross-section width of the sample, mm; *h* expresses the cross-section height, mm; and *d*(*t*) represents the creep deflection at the midspan with the change of time *t*, mm.

### 3.3. Numerical Simulation of Pure Asphalt Mortar

The viscoelastic behaviors of FRAM under the given fiber content can be obtained via laboratory tests. However, obtaining satisfactory results only by means of the laboratory test to understand the internal reinforced mechanism and flexural–rheological behavior at the different influence factors of the fiber is difficult. Therefore, after determining the constitutive behaviors of fibers and asphalt mortars, investigating the internal mechanism and analyzing the fiber effect with the help of a numerical simulation of fiber composite models are necessary.

First, an FE simulation of the control sample based on the viscoelastic constitutive model was implemented through a numerical analysis to verify the validity of the numerical modeling and material parameter input. Second, the FE simulation of fibers added into the mortar matrix based on the 3D random distribution fiber model referred in the literature [30] was conducted. The laboratory test was used to confirm the feasibility of the numerical modeling and the simulation of the fiber composites further. Finally, the influence of various fiber factors on the bending rheological behavior of asphalt mortars were discussed thoroughly and analyzed.

#### 3.3.1. Viscoelastic Parameters of Pure Asphalt Mortar

**Figure 4:**
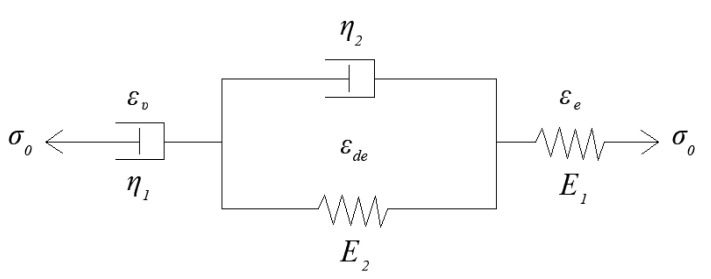
Burgers model.

The Burgers constitutive model obtained by combining basic viscoelastic elements was selected for asphalt mortar materials, as shown in Figure 4. The constitutive and creep strain equations are presented in Equations (3) and (4), respectively.

(3) $$\sigma +p_{1}\dot{\sigma }+p_{2}\ddot{\sigma }=q_{1}\dot{\varepsilon }+q_{2}\ddot{\varepsilon },$$

(4) $$\varepsilon (t)=\sigma_{0}[\frac{1}{E_{1}}+\frac{t}{\eta_{1}}+\frac{1}{E_{2}}(1-e^{\frac{E_{2}}{\eta_{2}}t})],$$

where $p_{1}=(\eta_{1}E_{1}+\eta_{1}E_{2}+\eta_{2}E_{1})/E_{1}E_{2}$, $p_{2}=\eta_{1}\eta_{2}/E_{1}E_{2}$, $q_{1}=\eta_{1}$, and $q_{2}=\eta_{1}\eta_{2}/E_{2}$; $E_{1}$ and $\eta_{1}$ represent the elastic modulus and viscosity index in the Maxwell model, respectively; $E_{2}$ and $\eta_{2}$ mean the elastic modulus and viscosity index in the Kelvin model, respectively.

According to the rheological values of the control sample obtained by laboratory tests, the four parameters of the Burgers constitutive model could be obtained through curve fitting, where the error on the measurements was within 10%. The fitted results of viscoelastic parameters for asphalt mortar at a flexural–tensile stress of 0.1 MPa were listed in Table 3, where the correlation coefficient *R*-square was 0.998. Given that the correlation coefficient of the fitted parameters was close to 1, the results indicated good fit. Hence, the parameters obtained using the control sample could be further used for the numerical simulation and analysis.

#### 3.3.2. Simulation of Flexural–Rheological Behaviors for Pure Asphalt Mortar

In the FE numerical simulation of the flexural–rheological values for the control sample model, the dimensions of the beam sample model were the same as those of the laboratory test. The 3D solid element was adopted, where the support span of the beam sample model was 120 mm. The international system of units (SI) was adopted (mm), where the units of length, force, time, and stress were mm, N, s, and MPa, respectively.

On the basis of the fitted results, four parameters of the control were further converted into the Prony series to characterize the viscoelastic properties in ABAQUS. The viscoelastic rheological parameters at 15 °C were presented in Table 4.

**Figure 5:**
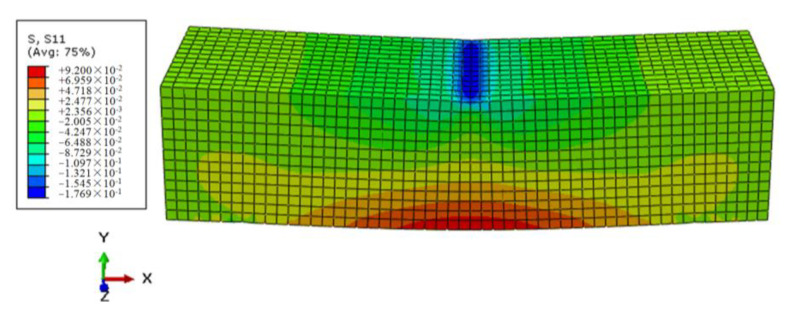
Stress contours of the control under a constant load.

**Figure 6:**
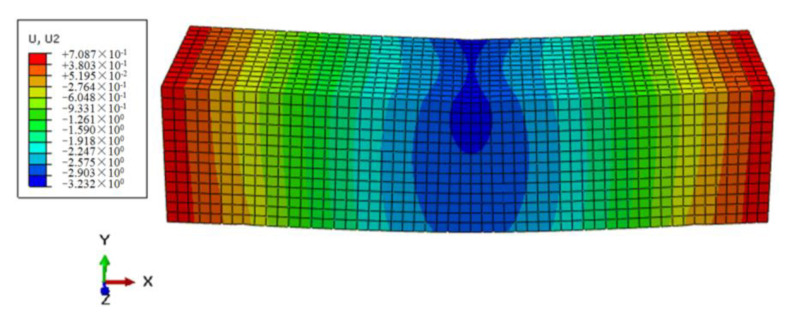
Vertical displacement contours of the control at 3600 s.

Through the process of repeated trial and error, the mesh size of the numerical model for the mortar matrix was set to 3, and C3D8 was selected as the element. A constant vertical load $F_{0}$ of 35.6 N was applied in the specimen midspan as an example, that is, the corresponding flexural–tensile stress $\sigma_{0}$ was 0.1 MPa. The simulation results of the control sample were shown in Figure 5 and Figure 6.

**Figure 7:**
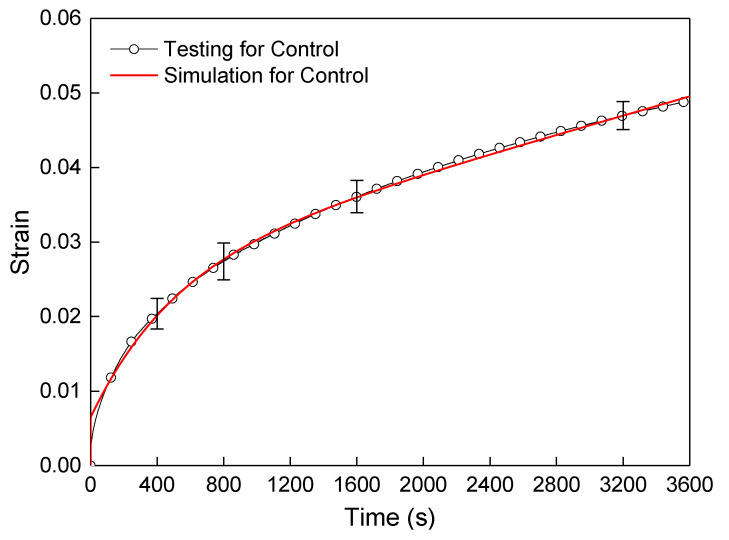
Comparison of flexural–tensile rheological curves for testing and simulation at 3600 s.

By observing the above contours of stress and displacement fields, the midspan vertical displacement for the control sample model could be got through post-processing, and then the strain of the beam midspan bottom can be obtained by Equation (2), and the calculated strain results were compared with testing values, as shown in Figure 7. It indicated that the simulated values of the control based on the Burgers constitutive model were consistent with the testing results. Furthermore, It also proved the rationality of the numerical modeling and the setting of material parameters. Furthermore, the simulation method could be further used in the modeling and analysis of the FRAM sample.

### 3.4. Numerical Simulation of FRAM

#### 3.4.1. Generation of A 3D Beam Sample Model for Fibers

The directional distribution of short fibers in asphalt-like material is an ideal assumption, so it is important to think about the actual distribution. In this study, a generation algorithm of the 3D random-distribution for short fibers in the cuboid matrix was proposed based on previous studies [29,35,36]. Furthermore, a program was created using the MATLAB software to establish a numerical model in FE software.

(a) Generation of random numbers

Usually, a recursive equation *X*_n+1_ = *R*(*X*_1_, *X*_2_, … *X*_n_) is used to generate random numbers, where *R* is the recursive function, and the new random number can be derived from the initial parameter values (*X*_1_, *X*_2_, …, *X*_n_). In the algorithm, the random number list {*X*_n+1_} is determined by the parameter values (*X*_1_, *X*_2_, … *X*_n_) and the function *R*. Thus, the random number list {*X*_n+1_} cannot completely meet the randomness and independence. In other words, the algorithm can get the same {*X*_n+1_} when the amount of *X*_n+1_ is sufficiently large. Therefore, {*X*_n+1_} is also called a pseudo-random number list. When the random number is small, the number list {*X*_n+1_} can meet the randomness completely [30]. In this study, the internal function *Rand* () in MATLAB was called directly to generate the number list {*X*_n+1_} on the basis of the generated principle of pseudo-random numbers. 

(b) Algorithm of the 3D random-distribution model

The fibers are considered as the random distribution in the asphalt mortar matrix, namely, the location and orientation of fibers are random. The total number of fibers is determined by the volume content *ρ_v_*, length *L*, and diameter *D* of the straight-round fiber in the mortar sample. The generated algorithm of the 3D model for fibers is described as follows [29,30,36]:

(1) A *Rand* () function is adopted to generate a random number.

(2) The total number of fibers in the mortar sample is calculated.

The volume *V_ρ_* of a single fiber is got based on the dimensions of fibers, and then the total volume of all fibers is calculated based on *ρ_v_* of total fibers in the sample model. Moreover, the total number of fibers in the 3D model can be obtained by the equation *V*·*ρ_v_*/*V_ρ_*, where *V* is the volume of the sample model.

(3) The random orientations and locations and of all fibers in the sample model are generated.

For the random orientation of fibers in the beam sample model, it can be calculated according to the algorithm of random rotation, where F_original and F_final represent the original and final orientations of the fiber; and *α*, *β*, and *γ* mean the angles rotating around the *X*, *Y*, and *Z* coordinate axes, respectively. The algorithm is as follows [30,36]:

(5) $$F\_\mathrm{final}=F\_\mathrm{original}*\left[\begin{array}{ccc} \cos\beta \cos\gamma & \cos\beta \sin\gamma & -\sin\beta \\ \sin\alpha \sin\beta \cos\gamma -\cos\alpha \sin\gamma & \sin\alpha \sin\beta \sin\gamma +\cos\alpha \cos\gamma & \sin\alpha \cos\beta \\ \cos\alpha \sin\beta \cos\gamma +\sin\alpha \sin\gamma & \cos\alpha \sin\beta \sin\gamma -\sin\alpha \cos\gamma & \cos\alpha \cos\beta \end{array}\right]$$

For the random location of fibers in the sample model, it can be calculated according to a random point F_Random (*X*_r_, *Y*_r_, *Z*_r_) and the initial location F_Origin (*X*_o_, *Y*_o_, *Z*_o_) of the fiber, where F_New (*X*_n_, *Y*_n_, *Z*_n_) is the new random location of the fiber in the model. The fiber is put into the model according to the following modified algorithm [30]:

*X*_n_ = *X*_o_ + *X*_r_;  *Y*_n_ = *Y*_o_ + *Y*_r_;  *Z*_n_ = *Z*_o_ + *Z*_r_ (6)

(4) The fibers are placed into the 3D model one by one and checked to ensure that all the location of the fibers are within the domain of the beam sample model.

The first fiber is positioned in the model according to the algorithm of random location and orientation and the boundary condition (fiber should not go beyond the model boundary). The nth fiber is then put in the model and it is ensured that the nth fiber does not intersect with and superpose the former (*n*–1) fibers. The distance between the centers of two fibers is not less than the fiber diameter *D*. The process continues until the last fiber is positioned.

(5) The location and orientation datum of fibers at given fiber contents are generated.

In the proposed 3D beam sample model, the location and orientation were obtained by two endpoints of the fiber. The model datum with INP file format generated by MATLAB were imported into FE software.

#### 3.4.2. Conversion of Fiber Contents

The weight percentage of fibers in the asphalt mortar can be calculated on the basis of the relationship of the asphalt mortar to corresponding mixture. Meanwhile, the fiber distribution in the asphalt mixture was assumed to be distributed completely in the asphalt mortar matrix. In this study, fiber contents are represented by the weight of mixture in the following statement, where the relationship of different fiber contents between the asphalt mixture and the corresponding mortar were shown in Table 5.

The total number of fibers should be obtained before conducting the mesostructural modeling and the creep simulation of the FRAM beam sample. Given that the total volume of fibers was extremely small in the FRAM sample, the total number of fibers can be obtained using the ratio of the total fiber weight to a single fiber. In this ratio, the total fiber weight corresponds to the weight percentage of the asphalt mixture, and the single fiber weight can be obtained from the fiber dimensions and its density. In this paper, the weight of fibers in the asphalt mixture with 0.3% fiber contents corresponded to approximately 5 g in the FRAM sample. For example, according to the BF size of 6 mm, 0.02 mm, and 2.7 g/cm^3^ in length, diameter, and density, respectively, the total number of 0.79% BF contents in asphalt mortar was approximately 982,500. Given that the number of fibers was close to a million, the fiber generation and the numerical calculation in the 3D composite model was difficult. However, the actual distribution of the fibers in the matrix was randomly dispersed, that is, it experienced clumping. Therefore, each fiber generated in the matrix is infeasible based on the 3D random distribution fiber model in Reference [30]. For simplicity, fibers bound as a fiber bundle are embedded in the mortar matrix to save time in the generated process of the total fiber number via the MATLAB code [28,29,30].

**Figure 8:**
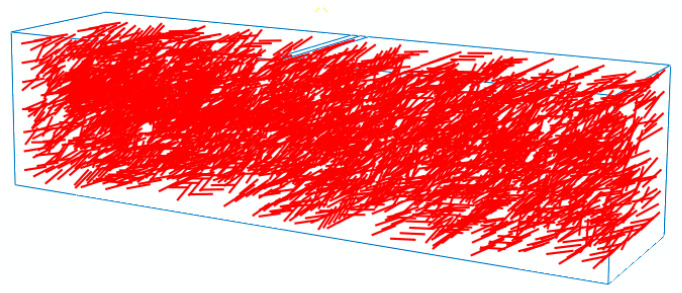
3D random-distribution fiber model.

To simplify the calculation and balance simulation precision, determining the fiber size and its number after fiber binding is necessary. In addition, the randomness of fiber distribution in the matrix must be considered as much as possible to ensure the sufficient quantity of fibers. Finally, after repeated trial and error calculations, obtaining the size of a fiber bundle with the length and diameter of 6 and 0.2 mm, respectively, is reasonable. For instance, when the fiber content was 0.3%, the number of BF and SWF fibers bound into one fiber bundle with a diameter of 0.2 mm under the same length of 6 mm were 225 and 4, and the corresponding numbers of the fiber bundle were about 9825 and 3890, respectively. The 3D random-distribution fiber model was shown in Figure 8.

#### 3.4.3. FE Modeling of FRAM

**Figure 9:**
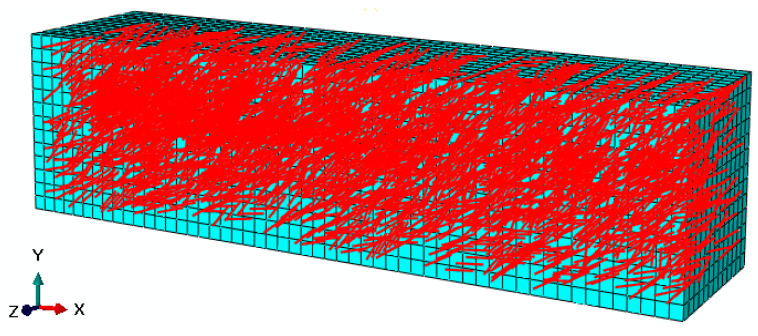
Finite element (FE) model for the FRAM sample.

In the FRAM composite model, the asphalt mortar polymer was considered a 3D solid element, and the fiber was modeled as a beam element. The minimum unit of the mesh was 3, and the element type was C3D8 for the matrix. The B31 element type was selected for fibers. A perfect adhesion between mortar polymer and fibers was assumed in this study. Figure 9 showed the generated typical 3D FE grids and the optimized analytical model for FRAM beam sample models in ABAQUS.

## 4. Results and Analysis

### 4.1. Effect of Fiber Contents

**Figure 10:**
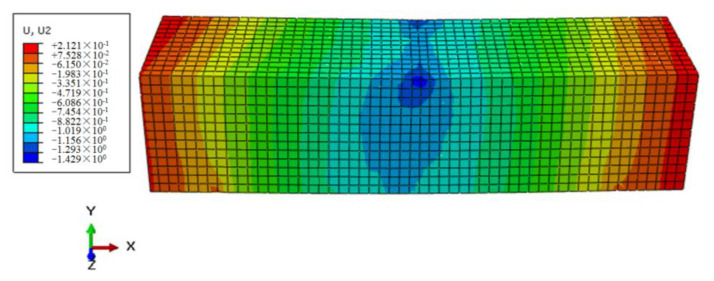
Displacement contours of the 3D model at 0.3% BF content at 3600 s.

Based on the 3D mesostructural model, the creep displacement of the FRAM beam sample under 0.3% BF content was simulated, and the vertical displacement contour at the flexural–tensile control stress of 0.1 MPa was presented in Figure 10.

**Figure 11:**
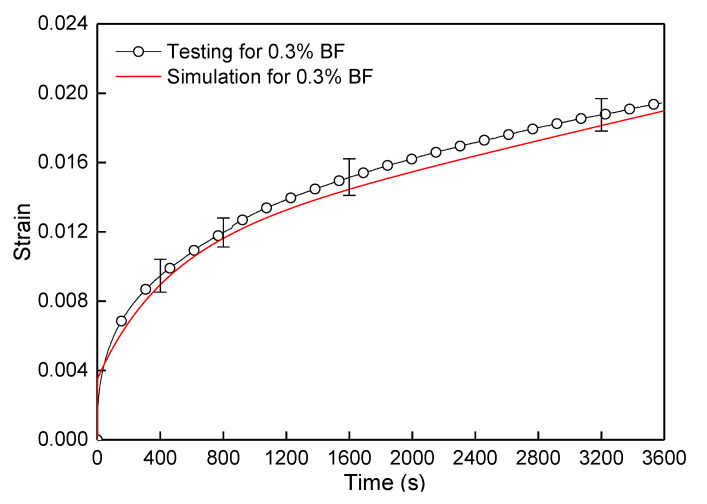
Rheological values of simulation and testing under 0.3% BF content.

Compared with the midspan vertical displacement of the control sample at 3600 s in Figure 6, the result of the FRAM sample in Figure 10 was significantly reduced after BF was added into the asphalt mortar. To compare the rheological values of the simulation and the testing results, the vertical displacement was converted into the rheological strain. Figure 11 shows that the rheological simulation value of FRAM under 0.3% BF contents was close to that of the test, where the deviation of the strain value at 3600 s was only 2.4%. Therefore, this finding can indicate that the random distribution algorithm of fibers was reliable, and the 3D numerical model of the FRAM sample was reasonable. The numerical model could be used to carry out simulation and analysis of the different fiber effects.

**Figure 12:**
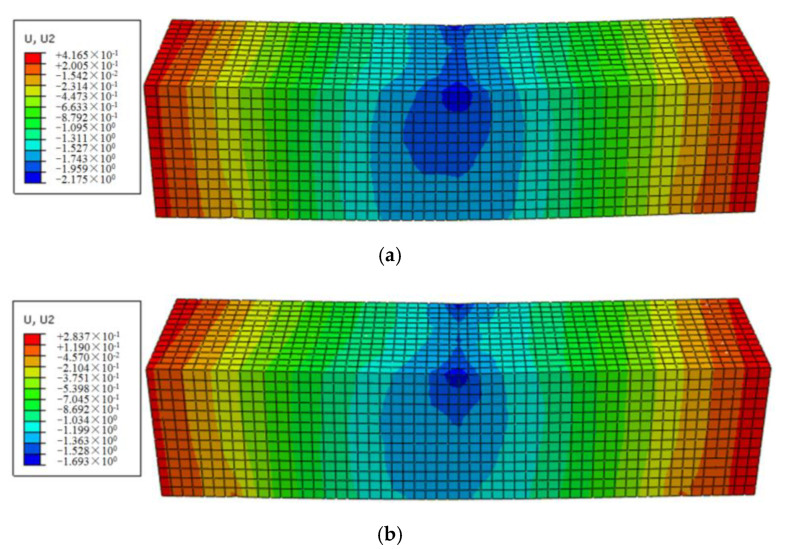
Displacement contour of the 3D model under different BF contents at 3600 s as: (**a**) 0.1% fiber content; (**b**) 0.2% fiber content.

**Figure 13:**
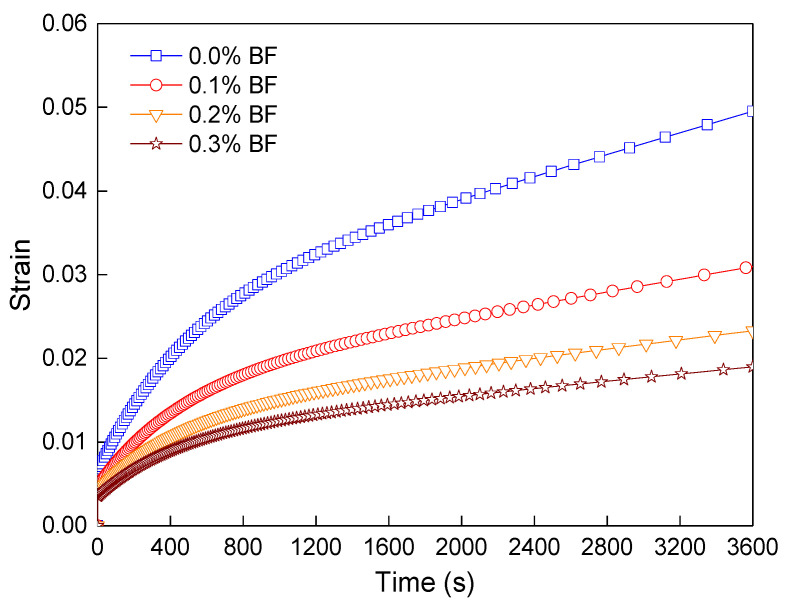
Flexural–rheological values of the 3D model under different BF contents.

Moreover, the influence of fiber contents on the bending rheological value and its property of asphalt mortars were investigated based on the 3D mesostructural model. The creep displacement of the asphalt mortar with 0.1% and 0.2% BF contents were further simulated, and its performance was analyzed. The vertical displacement contour was shown in Figure 12. The vertical displacement of the FRAM sample at the midspan was further converted into the flexural–rheological strain. The rheological values under different fiber contents at 3600 s were obtained by combining the results of Figure 11 and Figure 12, as shown in Figure 13.

Figure 13 indicates that the rheological value of FRAM decreased with the increase in BF contents, where the results of 0.1%, 0.2%, and 0.3% fiber contents compared with that of the control decreased by approximately 37.5%, 53%, and 61.7% at 3600 s, respectively. These findings also show that the increase in fiber contents within a certain range could effectively reduce the flexural–rheological strain of FRAM, thus improving the ability to resist the creep deformation of asphalt mortar.

**Figure 14:**
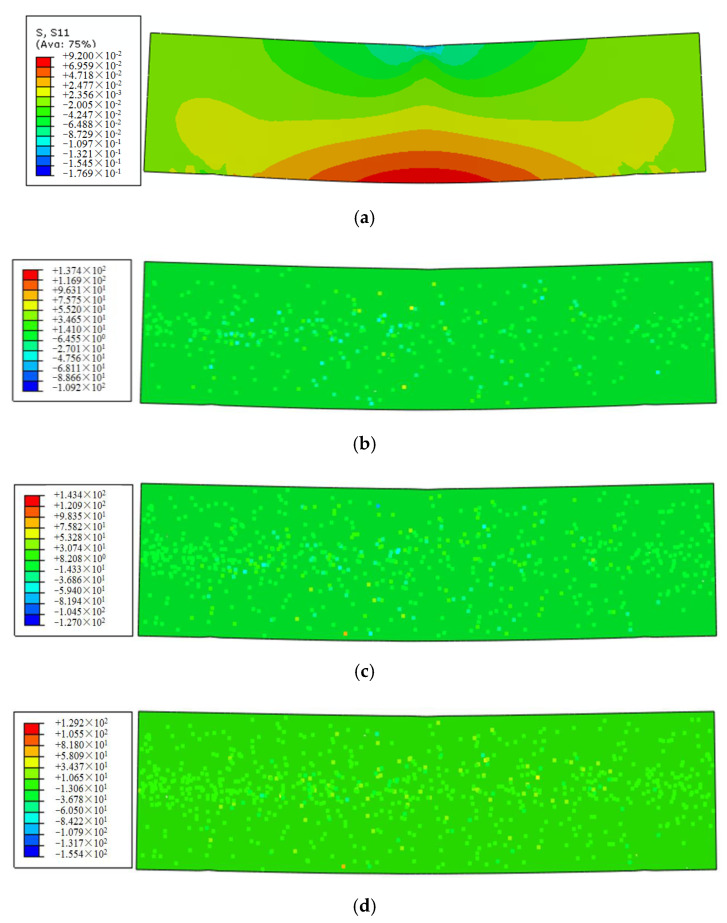
Stress contours of longitudinal section under different BF contents at 3600 s as: (**a**) the control; (**b**) 0.1% fiber content; (**c**) 0.2% fiber content; (**d**) 0.3% fiber content.

**Figure 15:**
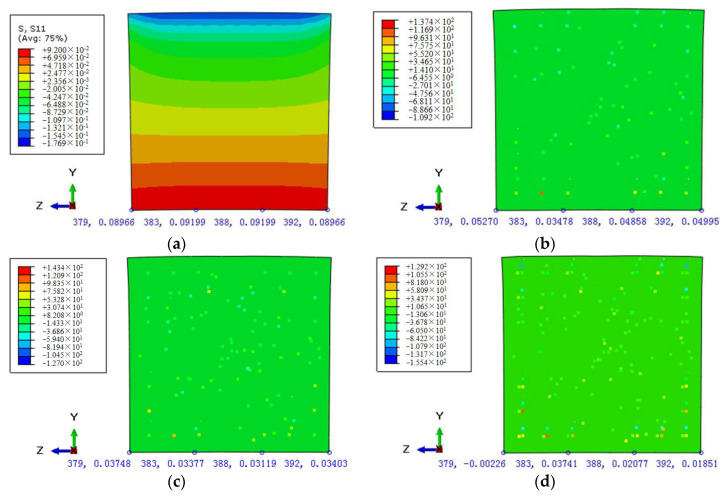
Stress contours of the midspan cross section under different BF contents at 3600 s as: (**a**) control; (**b**) 0.1% fiber content; (**c**) 0.2% fiber content; (**d**) 0.3% fiber content.

To explore the action mechanism of fiber further in the asphalt mortar under different fiber contents, the stress fields *S*_11_ of the samples along the longitudinal and traverse sections of the *x*- and *z*-axes were analyzed, respectively. The stress contours of the longitudinal and cross sections were illustrated in Figure 14 and Figure 15, respectively.

As shown in Figure 14a, the stress distribution of the longitudinal section for the control sample presents a clear gradient change at the midspan position, that is, the stress gradually transferred from the compression state at the top of the section to the tension state at the bottom. Furthermore, the FRAM samples shown in Figure 14b–d demonstrated that the stress field of the longitudinal section was redistributed after the addition of the BF into the mortar matrix. Hence, the stress value of the matrix was reduced, whereas that of the fiber was concentrated.

To conduct the quantitative analysis on the stress change at the control point in the midspan of the FRAM sample, four representative nodes (node numbers 379, 383, 388, and 392) at the cross-section bottom of the midspan were selected, and the mean values of flexural–tensile stress of such four nodes were compared under different BF contents. The stress contours of the cross section for the FRAM samples were shown in Figure 15. 

The comparison of the cross-section stress values between the control (Figure 15a) and the FRAM samples (Figure 15b–d) show that the mean value of flexural–tensile stress was significantly reduced with the increase in fiber contents, where the stress mean value of the FRAM sample for 0.1%, 0.2%, and 0.3% BF contents compared with that of the control was decreased by 48.4%, 62.3%, and 77.7%, respectively. Therefore, this finding thoroughly explains the phenomenon of the gradual decrease in flexural–tensile rheological strain with the increasing fiber content and BF with a significant reinforcement effect.

### 4.2. Effect of Fiber Aspect Ratios

**Figure 16:**
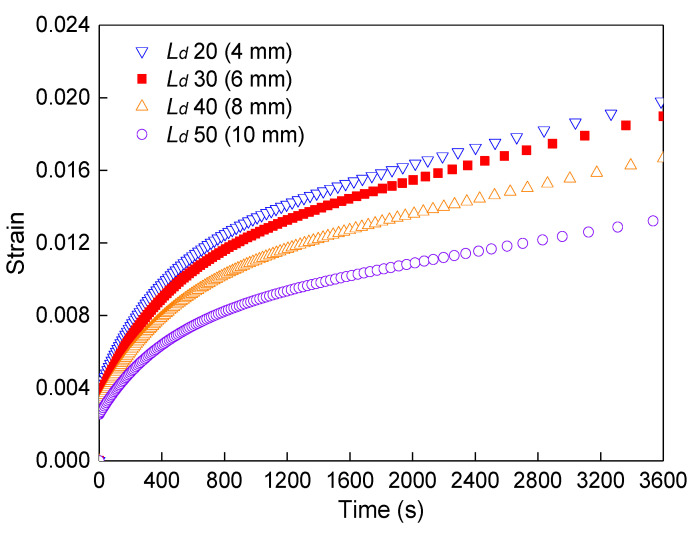
Flexural–tensile rheological values of the model under different BF aspect ratios.

After considering the flexural–tensile rheological value of FRAM under different fiber contents, this study further investigated the influence of different BF length–diameter ratios on the rheological value of the FRAM sample under 0.3% BF content. The size of diameter *D* for the fiber bundle was the same as that above, and the different fiber lengths *L* of approximately 4, 6, 8, and 10 mm were considered under the same diameter, whereby the fiber aspect ratios *L_d_* were set to 20, 30, 40, and 50, respectively. The simulated results of the flexural–tensile rheological strain for the FRAM sample under different fiber aspect ratios at 3600 s were demonstrated in Figure 16, where the flexural–tensile control stress was set to 0.1 MPa.

The rheological strain gradually reduced with the increase of *L_d_* value in the 3600 s loading, where the rheological values of 50 and 40 in *L_d_* compared with the result of 30 in *L_d_* reduced by 29.9% and 12.3%, respectively. Moreover, the result of 20 in *L_d_* compared with that of 30 in *L_d_* increased by 4.5%. Furthermore, the increase in the fiber length under the same diameter could effectively increase the flexural–tensile rheological properties or reduce the rheological strain, this is because fibers absorb more stress with the increase in fiber aspect ratios to make the mean stress value of the mortar matrix at the bottom of the beam midspan reduced, thus improving the fiber reinforcement effect. However, the increase in fiber length in actual engineering will cause difficulty in the uniformity of fiber mixing in the asphalt mixture and may result in clumping and difficulty in achieving the reinforcement effect. Therefore, considering the construction and control of the fiber length is also necessary.

### 4.3. Effect of Fiber Types

**Figure 17:**
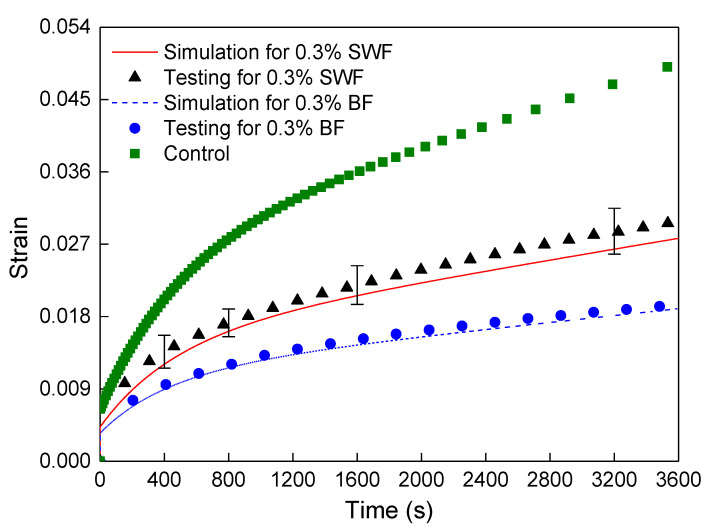
Flexural–tensile rheological values of the model under different fiber types.

This study also considered the influence of fiber types on the rheological value of FRAM, and the BF and SWF with 0.3% fiber content were selected. The length of the SWF was 6 mm, and the diameter of a single fiber was 0.1 mm. The numerical modeling process of the SWF was similar to that of BF and was more reasonable for simulation by using the trial calculation of binding four fibers into a fiber bundle with a diameter of 0.2 mm. Given that the density of the SWF (7.5 g/cm^3^) was higher than that of the BF (2.7 g/cm^3^), the number of the SWF bundle was smaller than that of the BF under the same fiber content. Hence, the result of the SWF bundle was 3890 under 0.3% fiber content. By simulating and combining the results with the laboratory test for SWF mortar under 0.1 MPa control stress, the rheological values of the numerical simulation were close to that of testing, as shown in Figure 17.

Figure 17 shows that the flexural–tensile rheological value of FRAM can be significantly decreased by 44.0% at 3600 s after adding the SWF into asphalt mortar. Moreover, the level of decrease was less than that of the BF mortar (a decrease of approximately 61.7%). Therefore, the reinforcement of the SWF was not as effective as that of the BF. Nevertheless, the SWF still had a better resistance to the bending creep of asphalt mortar. Although the elastic modulus of the SWF was significantly larger than that of the BF, the diameter and density of the SWF were all significantly larger than those of the BF in the reinforcement effect of the SWF. Hence, the number of SWFs should be significantly reduced under the same content to ensure that this reinforcement effect was slightly less than that of the BF.

### 4.4. Sensitivity Analysis of Viscoelastic Properties for FRAM

The viscoelastic creep curves of the control and FRAM were fitted to get the four parameters of the Burgers constitutive model on the basis of simulated results of the 3D mesostructural model and verification of the macroscopic laboratory tests discussed above. In this study, the BF was taken as an example to analyze the effect of fiber factors (e.g., fiber content, modulus, and aspect ratio) on the sensitivity of the Burgers constitutive parameters for FRAM.

**Figure 18:**
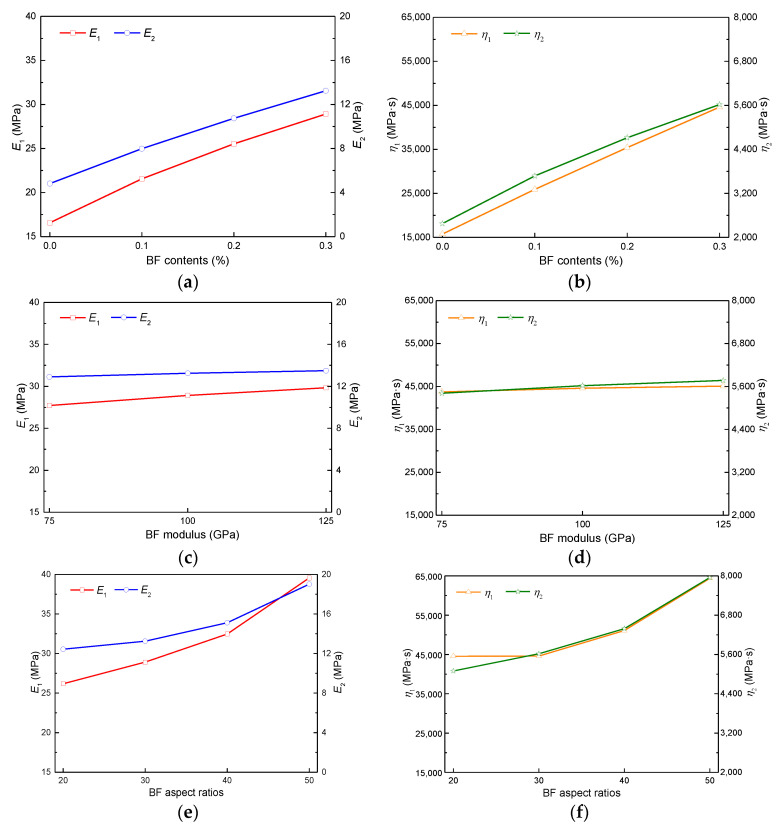
Relationship of BF factors to viscoelastic parameters for FRAM as: (**a**) E_1_ and E_2_; (**b**) η_1_ and η_2_; (**c**) E_1_ and E_2_; (**d**) η_1_ and η_2_; (**e**) E_1_ and E_2_; (**f**) η_1_ and η_2_.

The specific results regarding the effect of BF factors on the viscoelastic parameters of FRAM (Figure 18), the BF contents were selected as 0%, 0.1%, 0.2%, and 0.3%; BF modulus was set as 75, 100, and 125 GPa under 0.3% fiber contents and 30 aspect ratios; and BF aspect ratios were obtained as 20, 30, 40, and 50 under 0.3% fiber contents and 100 GPa modulus.

Figure 18 shows that the constitutive parameters increase with the increase of BF contents, modulus and aspect ratios, where the relationship between the four parameters and fiber contents, aspect ratios did not increase linearly, namely, the growth rate of parameters decreased gradually with the increase of fiber contents, and increased with the increase of fiber aspect ratios. However, the relationship between the four parameters and fiber modulus was approximately linear, and the sensitivity of the four parameters for fiber modulus was not as significant as that for fiber contents and aspect ratios. Based on the regression analysis of the above results, the unified prediction equation between Burgers parameters and fiber factors can be established, as follows

*P*(*a*, *b*, *c*) = *a^k^*^1^ × [*k*_2_ + *k*_3_ × (*b*/100-1) + *k*_4_ × (*e*^(*c*/30-1)^-1)] + *P*_0_ (7)

where *a*, *b*, and *c* represent fiber contents (%), modulus (GPa) and aspect radios, respectively; *P* is the parameter of Burgers model; *k*_1_, *k*_2_, *k*_3_, and *k*_4_ are coefficients of the equation and *P*_0_ is the Burgers parameter of the control.

The curve data in Figure 18 was fit by Equation (7), and the coefficients of prediction equations for the four parameters can be obtained by considering various factors as follows

*E_1_*(*a,b,c*) = *a*^0.8186^ × [33.18 + 11.36 × (*b*/100-1) + 28.92 × (*e*^(*c*/30-1)^-1)] + 16.57, (8)

*E_2_*(*a,b,c*) = *a*^0.8863^ × [24.54 + 1.93 × (*b*/100-1) + 16.45 × (*e*^(*c*/30-1)^-1)] + 4.82, (9)

*η*_1_(*a*,*b*,*c*) = *a*^0.9491^ × [90473.1 + 4626.8 × (*b*/100-1) + 58,915.7 × (*e*^(*c*/30-1)^-1)] + 15,745.3, (10)

*η*_2_(*a*,*b*,*c*) = *a*^0.8289^ × [8823.7 + 1100.3 × (*b*/100-1) + 6350.5 × (*e*^(*c*/30-1)^-1)] + 2376.4, (11)

where the unit of *E*_1_, *E*_2_ is MPa; the unit of *η*_1_, *η*_2_ is MPa·s. The effects of *a*, *b*, and *c* on FRAM were assumed as independent of one another in the above equations. The correlation coefficient *R^2^* for those regression Equations (8)–(11) were 0.992, 0.914, 0.886, and 0.918, respectively.

## 5. Conclusions

The flexural–tensile rheological strain of the corresponding samples was investigated via the 3D numerical simulation of FRAM, and the rationality of the fiber mortar model was verified through laboratory testing. Furthermore, the effects of different fiber factors on the rheological strain of FRAM were discussed. The following conclusions can be drawn from this study.

(1) The flexural–tensile rheological strain of FRAM gradually decreased with the increase in fiber content and aspect ratio. The results of the BF content for 0.1%, 0.2%, and 0.3% at 3600 s compared with the control reduced by approximately 37.5%, 53%, and 61.7%, respectively. The results of the BF aspect ratio for 30, 40, and 50 compared with that for 20 reduced by approximately 4.3%, 16.1%, and 32.9%, respectively. 

(2) The reinforcement effect of the SWF was not as effective as that of the BF. However, the SWF can still improve the flexural–tensile rheological behavior of asphalt mortar, as the rheological value of the SWF mortar for AC13 gradation compared with the control reduces by nearly 44.0% at 3600 s.

(3) The viscoelastic parameters had a positive correlation with BF contents, modulus, and aspect ratios, and the sensitivity of the four parameters for BF modulus was not as significant as that for BF contents and aspect ratios. Furthermore, the equations of the relationship between each parameter of the Burgers model and the fiber factors are established.

## Figures and Tables

**Table 1:** Gradation of the asphalt mixture and corresponding mortar.

Sieve Size (mm)	Retained Weight Percentage of Gradation (%)					
	1.18	0.6	0.3	0.15	0.075	<0.075
Asphalt mixture for AC13	9.0	7.8	5.5	2.7	2.3	7.9
Asphalt mortar for AC13	25.6	22.2	15.6	7.7	6.5	22.4

**Table 2:** Material properties of the fibers.

Types	Elongation (%)	Elastic Modulus (GPa)	Tensile Strength (MPa)	Density (g/cm^3^)
BF	3.1	100	4500	2.70
SWF	6.2	200	1500	7.50

**Table 3:** Viscoelastic parameter values for the control sample.

Parameters	*E*_1_ (MPa)	*η*_1_ (MPa·s)	*E*_2_ (MPa)	*η*_2_ (MPa·s)	*R*-square
Values	16.6	1.57×10^4^	4.82	2.38 × 10^3^	0.998

**Table 4:** Parameter conversion values of the Burgers model for the control.

Parameters	E1 (MPa)	*E*_2_ (MPa)	*η*_1_ (MPa·s)	*η*_2_ (MPa·s)	*g*_1_	*g*_2_	*τ*_1_	*τ*_2_
Values	16.6	4.82	1.57 × 10^4^	2.38 × 10^3^	0.812	0.188	102	4.61 × 10^3^

**Table 5:** Relationship of fiber contents between asphalt mixture and corresponding mortar.

Types	Values of Fiber Contents		
Fiber contents in asphalt mixture (%)	0.1	0.2	0.3
Fiber contents in asphalt mortar (%)	0.26	0.53	0.79
